# Induction of temperate cyanophage AS-1 by heavy metal – copper

**DOI:** 10.1186/1471-2180-6-17

**Published:** 2006-02-24

**Authors:** Lee H Lee, Doris Lui, Patricia J Platner, Shi-Fang Hsu, Tin-Chun Chu, John J Gaynor, Quinn C Vega, Bonnie K Lustigman

**Affiliations:** 1 Department of Biology & Molecular Biology, Montclair State University, Montclair, NJ 07043, USA; 2 Department of Health Informatics, University of Medicine and Dentistry of New Jersey, Newark, NJ 07107, USA

## Abstract

**Background:**

It has been reported that some marine cyanophage are temperate and can be induced from a lysogenic phase to a lytic phase by different agents such as heavy metals. However, to date no significant reports have focused on the temperate nature of freshwater cyanophage/cyanobacteria. Previous experiments with cyanophage AS-1 and cyanobacteria *Anacystis nidulans *have provided some evidence that AS-1 may have a lysogenic life cycle in addition to the characterized lytic cycle.

**Results:**

In this study, the possible temperate *A. nidulans *was treated with different concentrations of heavy metal-copper. CuSO_4 _with concentrations of 3.1 × 10^-3 ^M, 3.1 × 10^-4 ^M, 3.1 × 10^-5 ^M and 3.1 × 10^-6 ^M were used to detect the induction of AS-1 from *A. nidulans*. The population of the host, unicellular cyanobacteria *Anacystis nidulans*, was monitored by direct count and turbidity while the amount of virus produced was derived from plaque forming units (PFU) by a direct plating method. The ratio of AS-1 release from *A. nidulans *was also determined. From these results it appears that AS-1 lysogenic phage can be induced by copper at concentrations from 3.1 × 10^-6 ^M to 3.1 × 10^-4 ^M. Maximal phage induction occurred at 6 hours after addition of copper, with an optimal concentration of 3.1 × 10^-6 ^M.

**Conclusion:**

Cu^2+ ^is a significant inducer for lysogenic cyanobacterial cells and consequently would be a potential control agent in the cyanobacteria population in fresh water ecosystems.

## Background

*Anacystis nidulans *is a rod-shaped, unicellular prokaryotic cyanobacterium and plays an important role in aquatic ecosystems as a primary producer. It is often used as an indicator for studying the toxic metabolic levels of heavy metals. Many heavy metal studies have been done using *A. nidulans *as the model system for the reaction of organisms to heavy metal stresses [1-10]. In freshwater environments, dense algal blooms of cyanobacteria are usually caused by nutrient enrichment (*i.e*., nitrogen and phosphorus) from sewage, agricultural fertilizers and industrial run-off into waterways [11]. Algal blooms are considered threat to the water system [11,12]. Cyanophage are viruses that infect cyanobacteria and are ubiquitous in both freshwater and marine environments. These phages play important roles in modulating cyanobacterial populations, affecting primary productivity, increasing water quality and may have a profound influence on global biogeochemical cycles [13,14]. Although the interaction between a cyanophage and its host organism is important in maintaining water quality in freshwater systems, little is known about how viruses regulate microbial mortality in natural waters. Recently, it was found that lysogenic infection was common in marine *Synechococcus *sp. [15]. Cyanophage infecting a single strain of marine *Synechococcus *sp. can reach 10^3 ^to 10^5 ^per ml in seawater [14,16-18]. Suttle and Chan [17] have estimated that between 5–15 % of marine *Synechococcus *cells were lysed by cyanophage daily. The discovery of a high abundance of viral particles (*ca*. 10^7 ^per ml) in natural waters [19,20] initiated the research on the ecological impact of the viral infection and lysis of marine microbes. They also provided evidence that viruses can affect microbial populations by either going through a lytic cycle, causing destruction of the host cell, or maintaining a lysogenic stage, in which the viral genome is inserted and maintained as the prophage in its host cell [18]. There was also evidence to suggest that seasonal changes can cause the prophage to enter a lytic cycle thus leading to the disappearance of algae blooms [21-23]. Lysogeny can also be induced to a lytic cycle by pollutants [24].

Although extensive research has been done on the interaction between cyanophage and cyanbacteria in marine systems, there are no significant reports that have focused on freshwater cyanophage/cyanobacteria interactions. The possibility of temperate AS-1 and lysogenic *A. nidulans *was suggested by Bisen *et al *[25], but there was no direct evidence provided. It has been reported that UV, mitomycin C and heavy metals such as copper, cadmium can induce the release of cyanophage in marine water [22,26]. In this study, different concentrations of copper sulfate were used to study a possible AS-1 lysogenic life cycle in addition to the previously characterized lytic cycle. Addition of copper sulfate led to a significant increase in phage production, a characteristic of an organism with a lysogenic life cycle. The study of lytic induction from temperate *A. nidulans *can provide a good model for studying the interaction between cyanophage and cyanobacteria in freshwater ecosystems.

## Results and discussion

**Figure 1:**
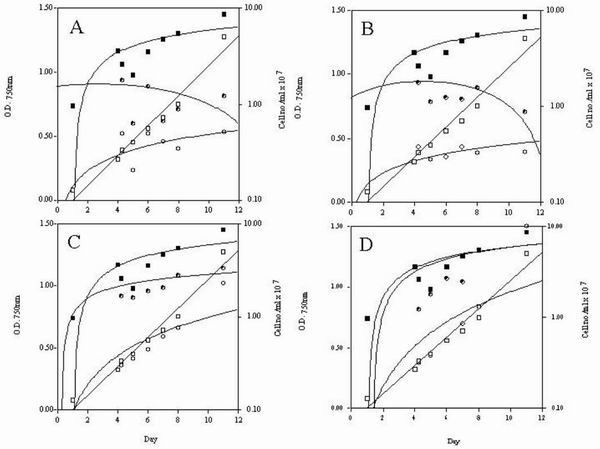
**The growth curve of AN-T and CuSO_4 _treated AN-T. **The AN-T growth curves with addition of different concentrations of CuSO_4 _at day 4, and no CuSO_4 _added as the control. A. Control and CuSO_4 _3.1 × 10^-3 ^M; B. Control and 3.1 × 10^-4 ^M; C. Control and 3.1 × 10^-5 ^M; D. Control and 3.1 × 10^-6 ^M. □ O.D. (Control); ■ Cell no./ml × 10^7 ^(Control); ○ O.D. (Treated); ● Cell no./ml × 10^7 ^(Treated).

In order to determine if heavy metals could cause induction of AS-1 from temperate *A. nidulans *(AN-T), CuSO_4 _was added at concentrations of 3.1 × 10^-3 ^M, 3.1 × 10^-4 ^M, 3.1 × 10^-5 ^M and 3.1 × 10^-6 ^M at day 4 post innoculation, the exponential growth stage of the culture. Growth of AN-T was severely inhibited at concentrations of 3.1 × 10^-3 ^M and 3.1 × 10^-4 ^M. Growth was affected to a certain extent in 3.1 × 10^-5 ^M of CuSO_4_; growth rate in 3.1 × 10^-6 ^M CuSO_4 _was very similar to the control (Figure 1).

**Figure 2:**
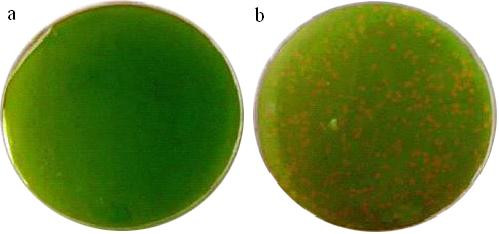
**PFU from CuSO_4 _induction. **Plaque Forming Unit (PFU) from AN-T after the CuSO_4 _induction; a) no plaques, b) proper numbers of PFUs for counting.

As results seen in marine cyanobacteria, there is consistent release of virus through out the lysogenic cycle in the normal AS-1/*A. nidulan*s infective system. The viral release was monitored by plaque forming units (PFUs). The representative pictures of the plaque forming units (PFU) after CuSO_4 _induction were obtained from different treated conditions and illustrated as no plaques and the proper number of plaques as showed in Figure 2. The results of AS-1 PFU study, showed that 6 hours after copper induction, 353.00 ± 32.57 PFUs were observed from the control, 483.00 ± 18.39 PFUs were observed from 3.1 × 10^-4 ^M of CuSO_4_; 416.00 ± 8.49 PFUs were formed from 3.1 × 10^-5 ^M of CuSO_4_, 480.00 ± 25.46 PFUs were formed from 3.1 × 10^-6 ^M of CuSO_4_. 24 hours after induction, 372.00 ± 16.97, 160.00 ± 11.31, 345.00 ± 18.39 and 526.00 ± 19.80 PFUs were formed compared to the control, 3.1 × 10^-4 ^M, 3.1 × 10^-5 ^M and 3.1 × 10^-6 ^M of CuSO_4 _respectively. 48 hours after induction, 479.00 ± 36.77, 57.00 ± 9.90, 483.00 ± 9.90 and 614.00 ± 5.66 PFUs were formed for the control, 3.1 × 10^-4 ^M, 3.1 × 10^-5 ^M and 3.1 × 10^-6 ^M of CuSO_4 _induction respectively.

PFUs per 10^6 ^*A. nidulans *was also calculated, 6 hours after induction, they were 13.57 ± 0.23, 27.44 ± 1.16, 24.83 ± 1.58, and 39.18 ± 0.41 respectively for the control, 3.1 × 10^-4 ^M, 3.1 × 10^-5 ^M and 3.1 × 10^-6 ^M of CuSO_4_. 24 hours after induction, they were 18.37 ± 2.47, 14.29 ± 0.44, 21.30 ± 1.24 and 29.06 ± 0.50 respectively for the control, 3.1 × 10^-4 ^M, 3.1 × 10^-5 ^M and 3.1 × 10^-6 ^M of CuSO_4_. 48 hours after induction, they were 13.42 ± 0.65, 4.67 ± 0.70, 25.76 ± 0.93 and 24.14 ± 0.21 respectively for the control, 3.1 × 10^-4 ^M, 3.1 × 10^-5^M and 3.1 × 10^-6 ^M of CuSO_4_.

The comparison of the treated AN-T with the control was summarized in Table 1. In the concentration of 3.1 × 10^-4 ^M, the PFUs were obvious at 6 hours after addition of copper; it induced 1.37 ± 0.08 (483.00 ± 18.39 / 353.00 ± 32.57) times the control. At 24 hours and 48 hours after addition of copper, the PFUs were significantly reduced, with only 0.43 ± 0.01 (160.00 ± 11.31 / 372.00 ± 16.97) times and 0.12 ± 0.01 (57.00 ± 9.90 / 479.00 ± 36.77) times of the control. The PFU per 10^6 ^*A. nidulans *with copper of 3.1 × 10^-4 ^M was 2.00 (27.44/13.57), 0.78 (14.29/18.37) and 0.35 (4.67/13.42) times of the control at 6, 24 and 48 hours respectively. With concentrations of 3.1 × 10^-5 ^M, PFUs were 1.18 ± 0.09 times (416.00 ± 8.49 / 353.00 ± 32.57), 0.93 ± 0.01 times (345.00 ± 18.39 / 372.00 ± 16.97) and 1.01 ± 0.06 times (483.00 ± 9.90 / 479.00 ± 36.77) of the control at 6, 24 and 48 hours respectively after the addition of copper. The PFU per 10^6 ^*A. nidulans *with copper of 3.1 × 10^-5 ^M was 1.80 (24.83/13.57), 1.20 (21.30/18.33) and 2.00 (225.76/13.42) times of the control at 6, 24 and 48 hours respectively after the addition of copper. With a concentration of 3.1 × 10^-6 ^M, the number of PFUs was 1.40 ± 0.06 times (480.00 ± 25.46 / 353.00 ± 32.57), 1.40 ± 0.01 times (526.00 ± 19.80 / 372.00 ± 16.67) and 1.28 ± 0.09 times (614.00 ± 5.66 / 479.00 ± 36.77) of the control at 6, 24 and 48 hours respectively after the addition of copper. The PFU per 10^6 ^*A. nidulans *with copper of 3.1 × 10^-6 ^M was 3.00 (39.18/13.57), 1.60 (29.06/18.37) and 1.80 (24.14/13.42) times of the control at 6, 24 and 48 hours respectively after the addition of copper. Concentrations of 3.1 × 10^-3 ^M may be too high to be inducers for releasing of temperate cyanophage AS-1 (Figure 1). This concentration of copper may be toxic for the growth of the cells and induce lethality through different mechanisms (4).

**Figure 3:**
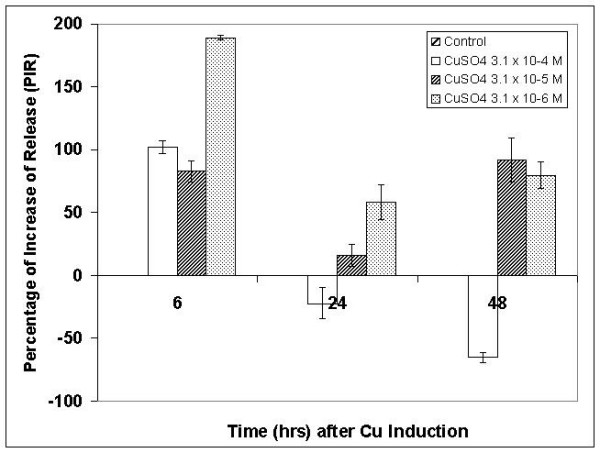
**Percentage of Increase of Release (PIR) from CuSO_4 _induction. **Heavy metal CuSO_4 _induction: PIR at 6 hours, 24 hours, and 48 hours after addition of different concentrations of CuSO_4 _at day 4 and non-CuSO_4 _added as a control.

From this study, the Percentage of Increase of Release (PIR) was also calculated for different conditions. The results indicated that 6 hours after addition of copper, 102.20 ± 5.19 %, 82.98 ± 8.61 % and 188.37 ± 1.80 % of increase of release were observed at 3.1 × 10^-4^M, 3.1 × 10^-5 ^M and 3.1 × 10^-6 ^M of CuSO_4 _respectively. 24 hours after the addition of copper, the induction efficiency was not of major consequence with 3.1 × 10^-4 ^M of CuSO_4_. The PIR was negative with a value of -22.20 ± 12.60 %. The PIR was 15.95 ± 8.79 % and 58.19 ± 14.00 % at 3.1 × 10^-5 ^M and 3.1 × 10^-6 ^M of CuSO_4 _respectively. 48 hours after addition of copper, the PIR for 3.1 × 10^-4 ^M of CuSO_4 _was -65.20 ± 3.65 %. The PIR was 91.95 ± 17.32 % and 79.88 ± 10.68 % at 3.1 × 10^-5 ^M and 3.1 × 10^-6 ^M of CuSO_4 _respectively (Figure 3).

While there is no clear evidence to explain why induction decreases over time, it is possible that either the phage/host interaction stabilizes after the initial stress or the toxic effect of heavy metal on the host causes a disruption in phage production.

Although the heavy metal induction rates varied depending on the concentrations of the heavy metal, the overall induction of copper compared to control is clear. The results suggest that Cu^2+ ^is a significant inducer for temperate AS-1 released from AN-T. The results correlated well with the study of induction for marine cyanobacterial lysogen although AS-1 release rate and induction rate by copper were much lower than the marine cyanophage/cyanobacterial lysogen studies [14,21,26]. Further study with other reported inducers mitomycin C and UV was also carried out to compare the PIR of both mitomycin C and UV with copper studies. The maximum PIR for different factors are showed in table 2. It is indicated that copper, UV and mitomycin C are able to induce the release of phage with PIR of 188.37 ± 1.80, 154.38 ± 15.00, and 162.86 ± 4.00 respectively.

## Conclusion

These results suggest that AS-1 lysogenic phage can be induced by copper with a concentration range from 3.1 × 10^-6 ^M to 3.1 × 10^-4 ^M. The best condition for phage induction occurred at 6 hours after addition of all these concentrations. Copper concentrations of 3.1 × 10^-6 ^M showed the highest level of viral induction. Cu^2+ ^is an important inducer for lysogenic cyanobacterial cells and consequently could be a potential trigger in the cyanobacteria population in freshwater aquatic environments.

## Methods

### I. Maintenance of cultures of *anacystis nidulans *and AS-1

#### 1. Culture and maintenance of *anacystis nidulans*

*Anacystis nidulans *was obtained from Dr. R. McGowan, Brooklyn College, N.Y. The culture was inoculated aseptically in a 250 ml Erlenmeyer flask with 100 ml Mauro's Modified Medium (3 M medium) at pH 7.9 [27]. The culture was grown in ambient temperature, with constant fluorescent light and continuous agitation at 100 rpm. Cell growth was monitored by direct cell count using a hemacytometer and turbidity studied using a Baush & Lomb Spectronic 20 at OD_750 nm _[13]. The cultures of *A. nidulans *were checked periodically for bacteria contamination by plating 100 μl of the culture on nutrient agar plates and observing after a 2 to 3 day incubation period. The stock cultures were maintained on 3 M agar plates and slants that were made with 3 M medium containing 2 % agar.

#### 2. Cultures, maintenance and titering of cyanophage AS-1

AS-1 was cultured aseptically in 250 ml Erlenmeyer flasks containing exponentially growing *Anacystis nidulans*. Sterile NaCl was added to the infected culture at a final concentration of 0.1 M. The flasks were gently shaken for 1 hour at room temperature to facilitate adsorption of the virus to the surface of the cell. The infected cultures were then incubated at room temperature under continuous cool-white fluorescent light. The growth of AS-1 was monitored by checking the lysis of the host cell. Host cell lysis was determined by turbidity studies using a Baush & Lomb Spectronic 20 at OD_750 nm _[28]. The lysis curve was generated by determining a decrease in the turbidity of the infected culture as well as by direct cell count using a hemacytometer as previously described.

The population of AS-1 was also monitored by plaque forming units (PFU). Pure non-viral infected *A. nidulans *(10 ml; AN-P) culture was centrifuged at 5,000 rpm for 10 minutes and the cell pellet was collected. At different time intervals, 2.5 ml were removed from cultures of temperate *A. nidulans (*AN-T) treated with different concentrations of copper and added into the cell pellet and mixed well. Melted 1 % 3 M soft agar (1 ml) was added to the mixture and vortexed. The mixture was then poured onto pre-warmed 2 % 3 M agar plates. After the soft agar solidified, the plate was placed under continuous "cool-white" fluorescent light for 5–7 days until the plaques (clear zone) were formed, and counted.

### II. Copper induction

Five ml of AN-T were inoculated respectively into 5 flasks containing 95 ml of 3 M medium to achieve a concentration of 1.0 × 10^7 ^cells/ ml. The cultures were grown for 4 days to reach exponential growth stage. Copper was then added to the cultures respectively using the following concentrations: CuSO_4 _3.1 × 10^-3 ^M, 3.1 × 10^-4 ^M, 3.1 × 10^-5 ^M, and 3.1 × 10^-6 ^M; a culture with no heavy metal was used as a control. The growth of AN-T was monitored by direct cell count using a hemacytometer and turbidity measured using a Baush & Lomb Spectronic 20 at OD_750 nm _for an 11 day period. The released AS-1 in the culture was monitored by the plaque plating method at 6 hours, 24 hours and 48 hours after addition of copper.

Percentage of Increase of Release (PIR)=(PFU per 106 cells of treated−PFU per 106 cells of controlPFU per 106 cells of control)×100%
 MathType@MTEF@5@5@+=feaafiart1ev1aaatCvAUfKttLearuWrP9MDH5MBPbIqV92AaeXatLxBI9gBaebbnrfifHhDYfgasaacH8akY=wiFfYdH8Gipec8Eeeu0xXdbba9frFj0=OqFfea0dXdd9vqai=hGuQ8kuc9pgc9s8qqaq=dirpe0xb9q8qiLsFr0=vr0=vr0dc8meaabaqaciaacaGaaeqabaqabeGadaaakeaacqqGqbaucqqGLbqzcqqGYbGCcqqGJbWycqqGLbqzcqqGUbGBcqqG0baDcqqGHbqycqqGNbWzcqqGLbqzcqqGGaaicqqGVbWBcqqGMbGzcqqGGaaicqqGjbqscqqGUbGBcqqGJbWycqqGYbGCcqqGLbqzcqqGHbqycqqGZbWCcqqGLbqzcqqGGaaicqqGVbWBcqqGMbGzcqqGGaaicqqGsbGucqqGLbqzcqqGSbaBcqqGLbqzcqqGHbqycqqGZbWCcqqGLbqzcqqGGaaicqqGOaakcqqGqbaucqqGjbqscqqGsbGucqqGPaqkcqGH9aqpdaqadaqaamaalaaabaGaeeiuaaLaeeOrayKaeeyvauLaeeiiaaIaeeiCaaNaeeyzauMaeeOCaiNaeeiiaaIaeeymaeJaeeimaaZaaWbaaSqabeaacqqG2aGnaaGccqqGGaaicqqGJbWycqqGLbqzcqqGSbaBcqqGSbaBcqqGZbWCcqqGGaaicqqGVbWBcqqGMbGzcqqGGaaicqqG0baDcqqGYbGCcqqGLbqzcqqGHbqycqqG0baDcqqGLbqzcqqGKbazcqGHsislcqqGqbaucqqGgbGrcqqGvbqvcqqGGaaicqqGWbaCcqqGLbqzcqqGYbGCcqqGGaaicqqGXaqmcqqGWaamdaahaaWcbeqaaiabbAda2aaakiabbccaGiabbogaJjabbwgaLjabbYgaSjabbYgaSjabbohaZjabbccaGiabb+gaVjabbAgaMjabbccaGiabbogaJjabb+gaVjabb6gaUjabbsha0jabbkhaYjabb+gaVjabbYgaSbqaaiabbcfaqjabbAeagjabbwfavjabbccaGiabbchaWjabbwgaLjabbkhaYjabbccaGiabbgdaXiabbcdaWmaaCaaaleqabaGaeeOnaydaaOGaeeiiaaIaee4yamMaeeyzauMaeeiBaWMaeeiBaWMaee4CamNaeeiiaaIaee4Ba8MaeeOzayMaeeiiaaIaee4yamMaee4Ba8MaeeOBa4MaeeiDaqNaeeOCaiNaee4Ba8MaeeiBaWgaaaGaayjkaiaawMcaaiabgEna0kabigdaXiabicdaWiabicdaWiabcwcaLaaa@CA09@

## Authors' contributions

LHL designed this study and supervised DL and SH at Montclair State University (MSU) and drafted the manuscript. DL, PJP, SH and TC carried out experiments; monitored the growth of AN, PFU of AS-1 and analyzed the data by ratio of release, times of induction and PIR. PJP and TC are research affiliates at MSU; they worked with DL and SH in the lab and drafted the manuscript. JJG, QCV, and BKL supervised DL, PJP, SH and TC and drafted the manuscript.

## Figures and Tables

**Table 1:** Summary of heavy metal CuSO_4 _induction. Heavy metal CuSO_4 _induction: Summary of the growth of AN-T; PFU; PFU per 10^6 ^*A. nidulans*; PFUs change rate for treated/control; PFU per 10^6 ^*A. nidulans *treated/control and PIR with different concentrations of CuSO_4 _at 6 hours, 24 hours, and 48 hours after addition of different concentrations of CuSO_4 _at day 4 and non-CuSO_4 _added as a control.

CuSO_4_	Control (No CuSO_4 _added)			
Time after induction (hrs)	0	6	24	48
OD _750 nm_	0.32 ± 0.03	0.39 ± 0.06	0.45 ± 0.03	0.56 ± 0.08
Cell numbers (10^7^)	3.60 ± 0.28	2.60 ± 0.27	2.06 ± 0.36	3.57 ± 0.10
Plaque Forming Unit (PFU)	ND	353.00 ± 32.57	372.00 ± 16.97	479.00 ± 36.77
PFU per 10^6 ^*A. nidulans*	ND	13.57 ± 0.23	18.37 ± 2.47	13.42 ± 0.65

CuSO_4_	3.1 × 10^-4 ^M CuSO_4_			
Time after induction (hrs)	0	6	24	48
OD _750 nm_	0.32 ± 0.03	0.44 ± 0.05	0.34 ± 0.01	0.36 ± 0.03
Cell numbers (10^7^)	3.60 ± 0.29	1.76 ± 0.14	1.12 ± 0.11	1.22 ± 0.03
Plaque Forming Unit (PFU)	ND	483.00 ± 18.39	160.00 ± 11.31	57.00 ± 9.90
PFU per 10^6 ^*A. nidulans*	ND	27.44 ± 1.16	14.29 ± 0.44	4.67 ± 0.70
PFUs change rate for treatment/control	ND	1.37 ± 0.08	0.43 ± 0.01	0.12 ± 0.01
PFU per 10^6 ^*A. nidulans *treated/control	ND	2.00	0.78	0.35
PIR (%)	ND	102.20 ± 5.19	-22.20 ± 12.60	-65.20 ± 3.65

CuSO_4_	3.1 × 10^-5 ^M CuSO_4_			
Time after induction (hrs)	0	6	24	48
OD _750 nm_	0.32 ± 0.03	0.36 ± 0.03	0.41 ± 0.04	0.49 ± 0.01
Cell numbers (10^7^)	3.60 ± 0.28	1.68 ± 0.14	1.62 ± 0.01	1.875 ± 0.11
Plaque Forming Unit (PFU)	ND	416.00 ± 8.49	345.00 ± 18.39	483.00 ± 9.90
PFU per 10^6 ^*A. nidulans*	ND	24.83 ± 1.58	21.30 ± 1.24	25.76 ± 0.93
PFUs change rate for treatment/control	ND	1.18 ± 0.09	0.93 ± 0.01	1.01 ± 0.06
PFU per 10^6 ^*A. nidulans *treated/control	ND	1.80	1.20	2.00
PIR (%)	ND	82.98 ± 8.61	15.95 ± 8.79	91.95 ± 17.32
CuSO_4_	3.1 × 10^-6 ^M CuSO_4_			
Time after induction (hrs)	0	6	24	48
OD _750 nm_	0.32 ± 0.03	0.38 ± 0.09	0.44 ± 0.04	0.56 ± 0.01
Cell numbers (10^7^)	3.60 ± 0.28	1.23 ± 0.08	1.81 ± 0.10	2.66 ± 0.13
Plaque Forming Unit (PFU)	ND	480.00 ± 25.46	526.00 ± 19.80	614.00 ± 5.66
PFU per 10^6 ^*A. nidulans*	ND	39.18 ± 0.41	29.06 ± 0.50	24.14 ± 0.21
PFUs change rate for treatment/control	ND	1.40 ± 0.06	1.41 ± 0.01	1.28 ± 0.09
PFU per 10^6 ^*A. nidulans *treated/control	ND	3.00	1.60	1.80
PIR (%)	ND	188.37 ± 1.80	58.19 ± 14.00	79.88 ± 10.68

PIR – Percentage of Increase of Release. ND – Not Detectable.

**Table 2:** Maximum percentage of increase of release (PIR) of copper, mitomycin C and UV. Comparison of maximum PIR of copper, mitomycin C and UV at different conditions is shown.

Factor	Conditions	Percentage of Increase of Release (PIR)
CuSO_4_	3.1 × 10^-6 ^M at 6 hours of induction	188.37 ± 2.00
UV	Exposure for 2.5 minutes	154.38 ± 15.00
Mitomycin C	0.5 μg/ml at 6 hours of induction	162.86 ± 4.00
